# Non-Coding and Coding RNAs in Targeted Cancer Therapy

**DOI:** 10.3390/cells15141264

**Published:** 2026-07-14

**Authors:** Macrina B. Silva-Cázares, César López-Camarillo

**Affiliations:** 1 Unidad Académica Multidisciplinaria Región Altiplano, Universidad Autónoma de San Luis Potosí, San Luis Potosi 78760, Mexico; macrina.silva@uaslp.mx; 2 Posgrado en Ciencias Genómicas, Universidad Autónoma de la Ciudad de México, Mexico City 03100, Mexico

Cancer remains one of the leading causes of morbidity and mortality worldwide despite remarkable advances in molecular biology, precision medicine, and targeted therapeutics. The increasing recognition that the majority of the human genome is transcribed into non-protein-coding RNAs has profoundly transformed our understanding of tumor biology. Once considered transcriptional noise, non-coding RNAs are now recognized as fundamental regulators of gene expression, cellular plasticity, metabolic adaptation, immune modulation, and therapeutic response. Together with protein-coding transcripts, these RNA molecules constitute intricate regulatory networks that determine cancer initiation, progression, metastasis, and resistance to treatment [1,2].

Recent years have witnessed an unprecedented expansion in RNA biology. MicroRNAs (miRNAs), long non-coding RNAs (lncRNAs), circular RNAs, small activating RNAs, antisense oligonucleotides, and other RNA-based molecules have emerged not only as biomarkers but also as promising therapeutic targets. Advances in transcriptomics, single-cell sequencing, spatial transcriptomics, and artificial intelligence-assisted bioinformatics have enabled the identification of increasingly complex RNA regulatory circuits that orchestrate tumor behavior across virtually every cancer type. Rather than functioning independently, coding and non-coding RNAs establish dynamic interaction networks that integrate transcriptional, post-transcriptional, epigenetic, and metabolic regulation [3,4,5,6].

The growing appreciation of RNA-mediated regulation has fundamentally altered the therapeutic landscape of oncology. Unlike conventional approaches that primarily target proteins, RNA-based strategies offer the possibility of intervening earlier in regulatory pathways, modulating multiple downstream signaling cascades simultaneously, and reaching targets previously considered undruggable. This paradigm shift has stimulated intense efforts to develop RNA therapeutics capable of selectively correcting aberrant gene expression while minimizing systemic toxicity [3,6,7,8].

One of the most compelling aspects of RNA biology is its remarkable versatility. Individual RNA molecules frequently participate in multiple cellular processes, serving as molecular scaffolds, decoys, guides, or competing endogenous RNAs that coordinate the activity of numerous signaling pathways simultaneously. Consequently, alterations in a single RNA species may propagate through extensive regulatory networks, influencing proliferation, apoptosis, angiogenesis, epithelial–mesenchymal transition, immune evasion, metabolic reprogramming, stemness, and metastatic dissemination [2,5,7].

Metabolic adaptation represents one of the defining hallmarks of malignant transformation. Tumor cells continuously rewire their metabolic pathways to sustain rapid proliferation, survive hypoxic conditions, and withstand therapeutic stress. While the Warburg effect remains a cornerstone of cancer metabolism, increasing evidence demonstrates that metabolic plasticity extends far beyond enhanced glycolysis. Cancer cells dynamically coordinate glycolysis, glutaminolysis, lipid metabolism, nucleotide biosynthesis, oxidative phosphorylation, and redox homeostasis according to microenvironmental demands. RNA molecules have emerged as essential regulators of this metabolic flexibility by controlling enzymes, transporters, transcription factors, and signaling pathways involved in nutrient sensing and energy production [1,4].

Beyond metabolism, RNA-mediated regulation also governs tumor interactions with the surrounding microenvironment. Cancer progression depends not only on intrinsic genetic alterations but also on reciprocal communication between malignant cells and stromal components, immune populations, endothelial cells, extracellular matrix, and soluble mediators. Non-coding RNAs actively participate in this bidirectional communication through intracellular signaling and extracellular vesicle-mediated transfer, contributing to immune suppression, angiogenesis, extracellular matrix remodeling, and metastatic niche formation. These observations reinforce the concept that RNA regulation operates at multiple biological levels, integrating intracellular programs with tissue-wide responses [2,4,5].

An additional hallmark receiving increasing attention is cellular plasticity. Tumor cells possess the extraordinary ability to dynamically transition between differentiated and stem-like phenotypes in response to environmental pressures. This plasticity underlies treatment resistance, tumor recurrence, and metastatic dissemination [2,5,7]. Non-coding RNAs play pivotal roles in regulating transcriptional programs that enable reversible phenotypic transitions, influencing epithelial–mesenchymal transition, cancer stem cell maintenance, lineage switching, and adaptation to therapeutic stress. Consequently, RNA-targeted interventions may provide opportunities to limit tumor evolution before resistant cellular populations become dominant [2,5].

Tumor vascularization also exemplifies the complexity of RNA-mediated regulation. While angiogenesis has traditionally been regarded as the principal mechanism supporting tumor growth, alternative vascularization processes have become increasingly recognized. Among these, vasculogenic mimicry illustrates the remarkable adaptability of aggressive tumor cells, which acquire endothelial-like characteristics and generate perfusable vascular-like channels independent of endothelial cells. This phenomenon contributes to tumor perfusion, metastatic dissemination, poor clinical prognosis, and resistance to anti-angiogenic therapies. Emerging evidence indicates that multiple non-coding RNAs coordinate transcriptional programs controlling extracellular matrix remodeling, hypoxia responses, epithelial–mesenchymal transition, and cellular invasion, thereby promoting vasculogenic mimicry across several malignancies. Understanding these regulatory networks may facilitate the development of therapeutic strategies capable of simultaneously targeting conventional angiogenesis and alternative vascularization mechanisms [1,2,4,5].

Equally transformative has been the development of RNA-based therapeutic technologies. Synthetic oligonucleotides, antisense molecules, RNA interference platforms, RNA editing systems, messenger RNA therapeutics, and CRISPR-associated technologies have expanded the range of possible molecular interventions. Improvements in chemical modification, nuclease resistance, stability, pharmacokinetics, and manufacturing have accelerated the translation of RNA therapeutics from experimental models to clinical applications. Several RNA-based drugs have already received regulatory approval, demonstrating the feasibility of manipulating gene expression in patients with acceptable safety profiles [3,6,8].

However, efficient and selective delivery remains one of the greatest challenges for RNA therapeutics. Naked RNA molecules are susceptible to rapid degradation, renal clearance, immune recognition, and limited intracellular uptake. Considerable efforts have therefore focused on developing delivery systems capable of protecting RNA molecules while ensuring tissue specificity and intracellular release. Lipid nanoparticles, polymeric carriers, extracellular vesicles, viral vectors, and ligand-mediated delivery systems have each contributed to overcoming these barriers. Among these approaches, ligand-directed targeting strategies offer particular promise because they combine molecular specificity with reduced systemic exposure, potentially improving therapeutic efficacy while minimizing adverse effects [6,8].

The convergence of RNA therapeutics with targeted delivery technologies represents an important step toward precision oncology. Rather than relying solely on tumor histology, future therapeutic decisions may increasingly incorporate transcriptomic signatures, RNA expression profiles, molecular subtypes, and patient-specific regulatory networks. Such personalized approaches could allow clinicians to identify patients most likely to benefit from RNA-directed therapies while simultaneously monitoring treatment response through minimally invasive liquid biopsy biomarkers [3,4,6].
